# Elevated Circulating Lactate Levels and Widespread Expression of Its Cognate Receptor, Hydroxycarboxylic Acid Receptor 1 (HCAR1), in Ovarian Cancer

**DOI:** 10.3390/jcm12010217

**Published:** 2022-12-27

**Authors:** Rachel Kerslake, Suzana Panfilov, Nashrah Mustafa, Marcia Hall, Ioannis Kyrou, Harpal S. Randeva, Emmanouil Karteris, Richard Godfrey

**Affiliations:** 1 Division of Biosciences, College of Health, Medicine and Life Sciences, Brunel University London, Uxbridge UB8 3PH, UK; 2 Mount Vernon Cancer Centre, Rickmansworth Road, Northwood HA6 2RN, UK; 3 Warwickshire Institute for the Study of Diabetes, Endocrinology and Metabolism (WISDEM), University Hospitals Coventry and Warwickshire NHS Trust, Coventry CV2 2DX, UK; 4 Warwick Medical School, University of Warwick, Coventry CV4 7AL, UK; 5 Research Institute for Health & Wellbeing, Coventry University, Coventry CV1 5FB, UK; 6 Aston Medical School, College of Health and Life Sciences, Aston University, Birmingham B4 7ET, UK; 7 Laboratory of Dietetics and Quality of Life, Department of Food Science and Human Nutrition, School of Food and Nutritional Sciences, Agricultural University of Athens, 11855 Athens, Greece; 8 Sport, Health and Exercise Sciences, College of Health, Medicine and Life Sciences, Brunel University London, Uxbridge UB8 3PH, UK

**Keywords:** lactate, HCAR1, biomarker, ovarian cancer (OvCa), high-grade serous ovarian cancer (HGSOC), liquid biopsy, screening

## Abstract

Background: Augmented glycolysis in cancer cells is a process required for growth and development. The Warburg effect provides evidence of increased glycolysis and lactic acid fermentation in cancer cells. The lactate end-product of glycolysis is receiving growing traction for its role as a cell signalling molecule. Ovarian cancer (OvCa) is also characterised by altered glucose metabolism. We aim to explore circulating lactate levels in patients with high-grade serous OvCa (HGSOC) and to elucidate the expression of the lactate receptor hydroxycarboxylic acid receptor 1 (HCAR1) in OvCa. Methods: HCAR1 expression was detected in patient biopsy cores using immunohistochemistry, while lactate was measured from whole blood with a Biosen-C line clinic measuring system. Results: We noted significantly elevated lactate levels in OvCa patients (4.3 ± 1.9 mmol/L) compared with healthy controls (1.4 ± 0.6 mmol/L; *p* < 0.0001), with an AUC of 0.96. The HCAR1 gene is overexpressed in OvCa compared to healthy controls (*p* < 0.001). Using an OvCa tissue microarray (>75% expression in 100 patients), high protein expression was also recorded across all epithelial OvCa subtypes and ovarian normal adjacent tissue (NAT). Conclusions: Lactate monitoring is a simple, cost-efficient test that can offer point-of-care results. Our data suggest that the potential of circulating lactate as a screening biomarker in OvCa merits further research attention.

## 1. Introduction

Lactate is produced when the rate of demand for adenosine triphosphate (ATP) is greater than what can be met through aerobic glycolysis alone. Hence, rather than pyruvate being converted to acetyl CoA and transported into the mitochondrion to enter the Krebs Cycle, pyruvate is reduced to lactate [1]. This results in a faster rate of production of nicotinamide adenine dinucleotide (NAD+) and adenosine triphosphate ATP in mammalian cells. The lactate produced here is the L-lactate isomer, whereas bacterial cells typically produce D-Lactate [2]. In humans, blood lactate concentrations are assessed routinely in sports sciences to establish the relative intensity of effort. Circulating lactate levels range from 0.2 to 2.3 mmol/L at rest in healthy individuals, and potentially rise to >25 mmol/L during maximal exercise [3,4].

In medical settings, lactate levels are routinely measured in critically ill patients; higher levels represent poorer tissue oxygenation and increased risk of death [5]. Conversely, cancer cells require high levels of energy and consequently have a lower threshold for undertaking glycolysis, even in the presence of adequate oxygen supplies [6]. As such, energy is often produced via the Embden–Meyerhof pathway earlier than in healthy cells under normoxic conditions [7,8]. This switch leading to lactate production is stimulated through the expression and activation of glycolytic enzymes including glucose transporters (GLUT) 1 and 3 [9]. Of note, GLUT1 is aberrantly expressed in many cancers, including ovarian cancer (OvCa) [10].

Overall, elevated glycolysis in cancer energy flux is relatively well established, while research has variously examined the role of lactate in cancer cell metabolism [6]. This pathway can drive an increase in cellular pH, mRNA expression of monocarboxylic acid transporters (MCTs), MCT1 and MCT4, as well as an increase in the catabolic enzyme, lactate dehydrogenase A (LDH) [11,12,13,14,15]. Moreover, lactate transport is also suggested to hold a concurrent role in cancer cell signalling; tumour cells with high glucose and hypoxic environments are thought to produce lactate, which is in turn metabolised by neighbouring tumour cells [16]. LDH is a catalytic enzyme involved in the reversible conversion of pyruvate to lactate during glycolysis. Increased levels of LDH are well documented in many cancer patients including those with melanoma, breast, lung, uterine and colorectal cancers [17,18]. In this context, LDH are used as part of the risk classifications for metastatic renal cell carcinoma and for non-Hodgkin’s lymphoma [17]. As with multiple other malignancies there is evidence that higher levels of LDH are found in patients with more advanced OvCa (FIGO Stage III/IV), and are associated with poor survival [13]. However, testing for LDH is relatively time-consuming, requiring a lab-based cytotoxicity assay (also known as an LDH release assay). In contrast, the measurement of blood lactate concentration is comparatively time-efficient and cost-effective and can be performed with point-of-care testing (e.g., at the outpatient examination room) [4].

Currently, an increasing body of evidence associates lactate signalling with multiple roles in the early development and progression of cancer via effects on tumour growth, angiogenesis, metastasis and immunosuppression [19]. In addition, lactate is seen to influence cytokine production through G protein-coupled receptor (GPCR) signalling [20]. Therefore, lactate’s biomarker and prognostic potential are currently under exploration [12]. As such, increased lactate production is now recognised as a pivotal step in early development of malignancy [21]. Further research on its role in the tumour microenvironment is therefore expected to further advance understanding of the underlying cancer biology. Moreover, emerging evidence suggests that lactate production contributes to carcinogenesis, supporting anabolic growth and proliferation in mechanisms outlined by the Warburg effect [6]. There is a plethora of studies on the Warburg effect published over the past decade. The Warburg effect describes how cancer cells alter their metabolism to favour growth and proliferation by increasing glucose uptake and fermenting it to lactate [8]. In line with this, circulating lactate levels 40 times the level of normal resting levels have been observed in head and neck tumours; with altered energy metabolism also being recognised as a hallmark of cancer [6,22].

Further supporting its potential implication in carcinogenesis is the fact that lactate is not just a by-product of altered metabolic reprogramming but is also implicit in signalling pathways through GPCR activation [6,23,24]. So far, it is well-known that the cognate receptor of lactate, hydroxycarboxylic acid receptor 1 (HCAR1; formerly known as GPR81), is a GPCR primarily expressed in adipose tissue, where its activation causes inhibition of lipolysis via a Gi-dependent pathway [25]. However, recently, elevated HCAR1 expression was also implicated in tumour growth and metastasis in cancers, such as breast and pancreatic [24,26]. Moreover, a study by Wagner et al. suggests that increased HCAR1 expression and activation in cervical cancer cells is capable of modulating cellular DNA repair mechanisms [27]. Additional silencing of HCAR1 may also down-regulate levels of BRCA1; a protein involved in DNA repair known for its mutagenic status in breast and OvCa [27,28].

Although elevated circulating lactate levels have already been documented in certain cancers (e.g., in breast, prostate and colorectal cancer), changes in circulating lactate are yet to be studied in patients with OvCa [29,30,31]; a malignancy characteristically associated with dysregulated energy metabolism and aberrantly expressed glucose [32]. This gynaecological malignancy has poor prognosis and usually remains undetected until late stages due to initial non-specific symptoms and the need for invasive examination, such as a transvaginal ultrasound [33]. As such, in the present study, we measured the circulating lactate levels of patients with OvCa, whist we further investigated the expression of its cognate receptor, HCAR1, at both gene and protein level, using in silico tools, tissue microarrays and whole blood lactate analysis techniques.

## 2. Materials and Methods

### 2.1. Blood Samples and Lactate Analysis

Blood samples from patients with high-grade serous ovarian cancer (HGSOC) (*n* = 53; all diagnosed with Stage III or IV) were collected from the Mount Vernon Cancer Centre, East and North Hertfordshire NHS Trust, as part of the CICATRIx study; 45 healthy adult women were also recruited as study controls.

The median age for patients (*n* = 53) and controls (*n* = 45) studied were 69 years (range 37–84) and 34 years (range 21–59), respectively. Control patients were volunteers who had no significant health concerns and were not on any treatment. All 53 patients had FIGO Stage III/IV HGSOC, and were being managed according to standard UK practice. This involves primary surgery followed by adjuvant chemotherapy for some and neo-adjuvant chemotherapy with interval or no surgery for others. Following chemotherapy (±surgery), patients generally receive maintenance targeted therapy with antiangiogenics (bevacizumab—Bev) and/or PARP inhibitors (PARPi). Blood samples were obtained at various points during treatment. Twenty-five patients had samples taken at diagnosis either prior to starting any treatment at all or after their primary surgery but prior to adjuvant chemotherapy treatment (PreC: *n* = 25). Twelve patients had samples taken when they were in clinical remission, after chemotherapy (maintenance Bev/PARPi). Finally, 16 patients had samples taken when they had shown evidence of relapse HGSOC, but prior to starting any further chemotherapy (Relapse OC-PreC).

Approximately 50% patients with ovarian cancer harbour homologous recombination repair deficiencies (HRD) [34]. Identification of BRCA wildtype OC patients with defective homologous repair has significant implications for prognosis and management. Current genomic methods, lack clinical validation and practical issues impede clinical implementation [35]. Given the importance of BRCA status in these patients, we further categorized the patients into BRCA wild-type and HRD negative (BRCAwt/HRD-ve, *n* = 40) or BRCA mutant group (germline or somatic) and HRD positive, as demonstrated by the Myriad MyChoice CDx test (BRCAmt/HRD+ve, *n* = 9).

The study was approved by the West Midlands–South Birmingham Ethics Committee (reference 16/WM/0196; protocol number RD2016-08). All participants provided written informed consent. Blood samples were collected either intravenously or via capillary lancet, with the study participants resting, at least 15 min prior to donation, to ensure standardized resting conditions. Blood (10 μL) was isolated using Accu-Chek Safe-T-Pro Plus Lancets (Roche, Switzerland), a sterile glass capillary tube (HaB International Ltd., Warwickshire, UK), and was mixed with 500 μL of haemolyzing solution (HaB International Ltd., Warwickshire, UK). Samples were inverted to mix before lactate analysis with a Biosen C line Clinic measuring system (EKF Diagnostics, Cardiff, UK).

### 2.2. Bioinformatic Analysis

CanSAR (cansar.icr.ac.uk, accessed on 10 August 2022), an integrative translational research and drug discovery knowledge base, was used to present HCAR1 expression across a range of cancers from The Cancer Genome Atlas (TCGA). GTEx (gtexportal.org/home/, accessed on 10 August 2022) was used to reveal normal gene expression in female reproductive tissues (cervix, fallopian tube, ovary, uterus, and vagina). GEPIA online tool (gepia.cancer-pku.cn/, accessed on 10 August 2022) allowed gene expression comparison between ovarian epithelial tissue (Genotype-Tissue Expression; GTEx) and OvCa biopsies (TCGA). Therapeutic response and survival rates of patients with OvCa were generated using Kaplan–Meier (KM) survival plots (www.kmplot.com, accessed on 10 August 2022).

### 2.3. Immunohistochemistry

Protein expression of HCAR1 in OvCa was assessed using immunohistochemical staining, following the methods outlined in our previous work [36]. An OvCa tissue microarray containing 90 OvCa and 10 normal ovarian biopsy samples was purchased from BioMax Inc. (Rockville, MD, USA) cat. No. BC1111d (Supplementary Table S1). Tissue samples were collected under Health Insurance Portability and Accountability Act (HIPAA) approved protocols and ethical standards. Unless otherwise stated, reagents were purchased from ThermoFisher Scientific (Waltham, MA, USA). Briefly, the array was deparaffinised and rehydrated, followed by antigen retrieval using sodium citrate solution (10 mM sodium citrate in dH_2_O, 0.05% Tween-20, pH 6.0) at 90 °C for 10 min. Washes in 0.025% Triton-X in PBS preceded 15 min incubation with 3% H_2_O_2_. The array was again washed prior to blocking in 5% BSA in PBS, before overnight incubation at 4 °C with HCAR1 primary antibody (1:100). Additional washes preceded an hour incubation with secondary antibody in 1% rabbit serum (ZytoChem Plus HRP-DAB Kit, Zytomed Systems, Berlin, Germany), before subsequent washes and 30 min incubation with streptavidin-HRP conjugate of the same brand and further washes. Finally, DAB stain and haematoxylin counterstain were applied followed by bluing with 0.1% sodium bicarbonate. The array was then dehydrated and sealed, before immunoreactivity quantification by the primary investigator and independent reviewers using a Leica light microscope (Zeiss, Oberkochen, Germany).

### 2.4. Statistical Analysis

Bioinformatic data were generated using open access online analytical tools with pre-set statistical methodologies with access to TCGA and GTEx data. GEPIA generated differential analysis was calculated using one-way ANOVA, taking gene expression of the normal (GTEx) against disease (TCGA), generating expression as log2(TPM+1). Expression is transformed for differential analysis with log2FC defined as median (Tumour)—median (Normal) with differentially expressed genes (DEGs), characterised as higher |log2FC| and lower q values, compliant with GEPIA’s pre-set threshold (http://gepia.cancer-pku.cn/help, accessed on 10 August 2022). Survival variances were generated using Kaplan–Meier plotter (kmplot.com, accessed on 10 August 2022). Statistical analyses for inhouse experiments were performed using GraphPad Prism9^®^ (v.9.4.1—GraphPad Software, Inc., San Diego, CA, USA). Error is represented using the standard error of mean (SEM). An ANOVA or *t*-test was applied to the data, based on parametric state and variable status. Unless stated otherwise, significance levels were set at *p* < 0.05. Receiver Operating Characteristics (ROC) curves were also generated using GraphPad Prism.

## 3. Results

### 3.1. Blood Lactate Levels Are Elevated in Patients with OvCa

**Figure 1:**
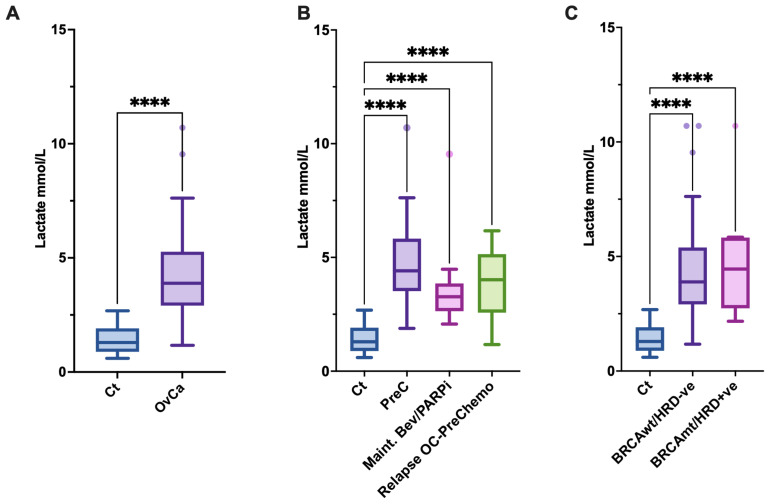
Lactate levels are elevated in OvCa. (**A**): Circulating lactate levels in controls (Ct; *n* = 45), patients with ovarian cancer (OvCa; *n* = 53); **** *p* < 0.0001. (**B**): Controls (Ct) compared to OvCa patients prior to any adjuvant chemotherapy treatment (Prechemotherapy, PreC), patients on maintenance bevacizumab, and or PARP inhibitors (Maint. Bev/PARPi), and those patients with samples taken prior to commencing chemotherapy treatment for HGSOC relapse (Relapse OC-PreChemo); **** *p* < 0.0001. (**C**): Controls (Ct) compared to OvCa patients with confirmed BRCA wild-type and HRD negative (BRCAwt/HRD-ve) versus patient carrying BRCA mutations or being identified as HRD positive (BRCAmt/HRD+ve); **** *p* < 0.0001.

Figure 1A demonstrates lactate levels (mmol/L) in the control (1.4 ± 0.6) vs. OvCa groups (4.3 ± 1.9), **** *p* < 0.0001. There were significantly higher lactate levels seen in all ovarian cancer patient groups when compared with the control group: Pre-Chemo (4.9 ± 1.9), Maintenance Bev/PARPi (3.7 ± 1.9), Relapse-PreChemo (3.8 ± 1.5) **** *p* < 0.0001. There were no differences between the BRCAwt/HRD negative (4.2 ± 1.8) and BRCAmt/HRD positive (4.9 ± 2.6) groups both of whom had similarly higher levels of lactate than the control group **** *p* < 0.0001.

**Figure 2:**
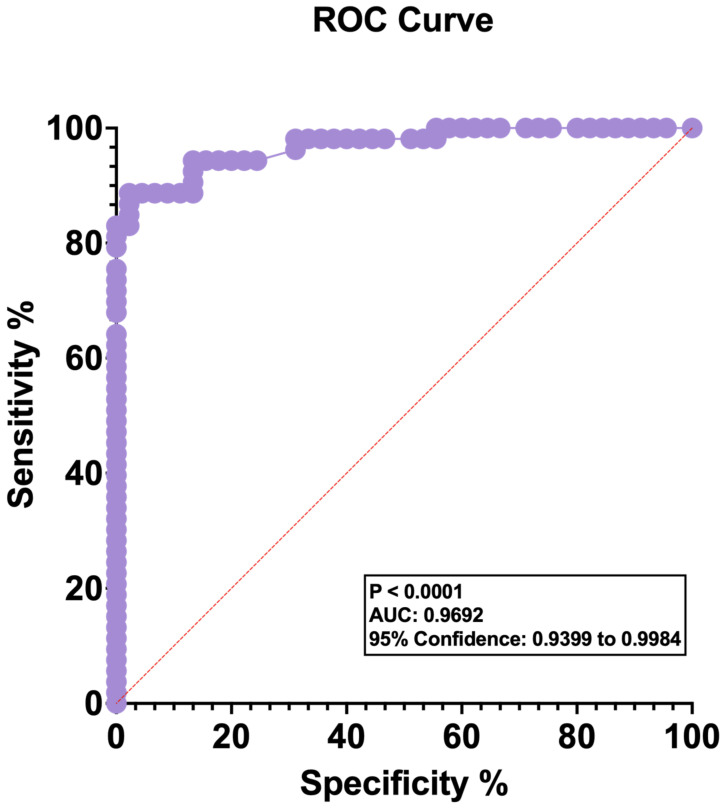
Receiver operating characteristic (ROC) plot. Patients with ovarian cancer (OvCa): *n* = 53; healthy female controls: *n* = 45. Area under the curve (AUC): 0.9692. The 95% confidence measuring between 0.9399 and 0.9984, *p* < 0.0001.

Figure 2 presents Receiver Operating Characteristic (ROC) plot depicting lactate concentration comparisons between OvCa patients and healthy controls. The data show an area under the curve of 0.96, with a high confidence interval (*p* < 0.0001). The specificity of lactate in OvCa therefore compliments sensitivity with a high level of accuracy.

### 3.2. Elevated Gene Expression of the Lactate Receptor HCAR1 in OvCa

**Figure 3:**
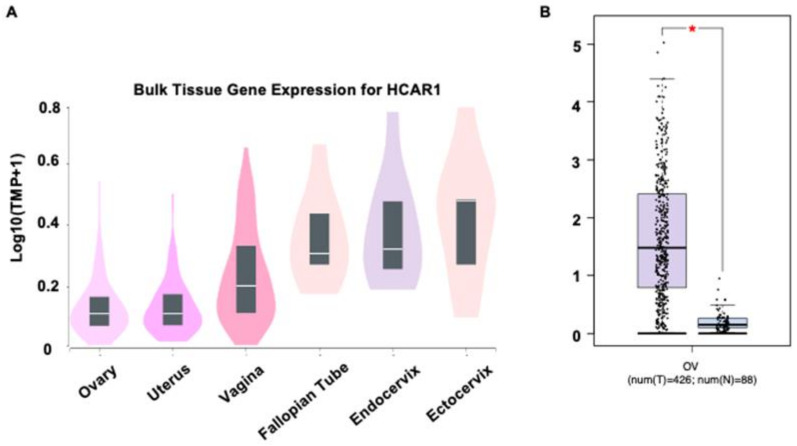
Expression of HCAR1 (hydroxycarboxylic acid receptor 1, HCAR1; formerly known as GPR81) in female reproductive tissues. (**A**) HCAR1 RNA expression in normal female tissues taken from the Genotype-Tissue Expression project (GETx); (**B**) HCAR1 expression in ovarian cancer (OvCa) tissue compared with normal ovarian epithelial tissues (OvCa = 426; *n* = 88), **^★^** *p* < 0.001. TPM: transcripts per million.

A series of in silico and lab-based approaches to map the gene and protein expression of HCAR1 in OvCa, were used. Using data from the public domain, GTEx, widespread expression of HCAR1 is seen in numerous cancers and stages (Supplementary Figure S1). Initial analyses of HCAR1 mRNA expression in a range of normal gynaecological tissues indicated that HCAR1 mRNA is present in cervix, fallopian tube, ovary, uterus, and vaginal tissues (Figure 3A). Expression of HCAR1 in OvCa tissue (*n* = 426) was significantly upregulated in comparison with normal ovarian epithelial tissue (*n* = 88) (Figure 3B).

### 3.3. High HCAR1 Protein Expression across Epithelial Subtypes of OvCa

**Figure 4:**
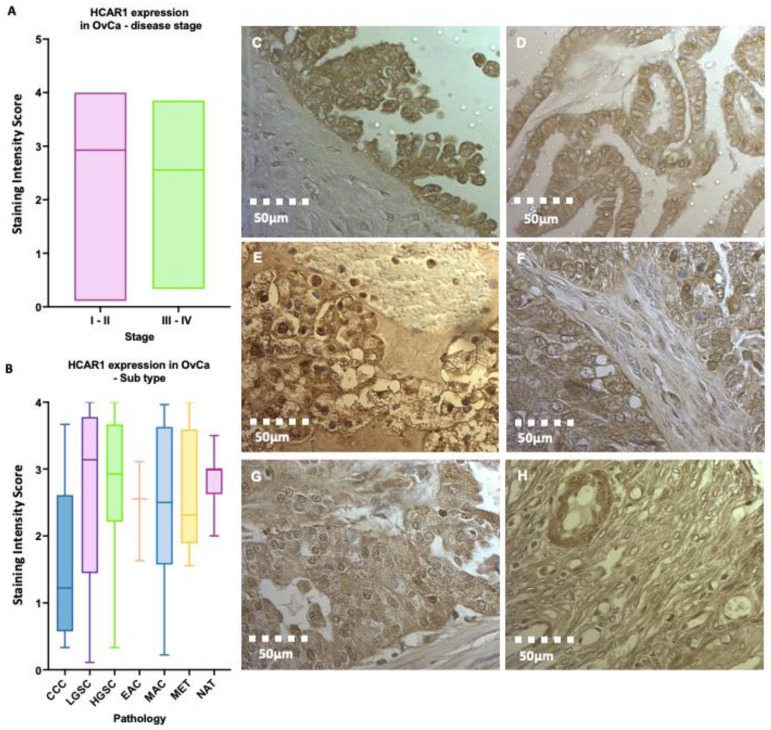
Expression of HCAR1 (hydroxycarboxylic acid receptor 1, HCAR1; formerly known as GPR81) in an ovarian tissue microarray containing biopsies from 90 patients with ovarian cancer (OvCa), in addition to 10 normal adjacent tissue (NAT) samples. (**A**) Intensity of tissue stained for early (I–II, *n* = 62) and late stage (III–IV, *n* = 18) OvCa, (no significance: ns); (**B**) Intensity of HCAR1 staining categorised by OvCa subtype: clear cell carcinoma (CCC), high-grade serous carcinoma (HGSOC), low-grade serous carcinoma (LGSOC), endometrioid adenocarcinoma (EAC), mucinous adenocarcinoma (MAC), lymph node metastasis (MET), and normal adjacent tissue (NAT). Quantification of cores was conducted by the primary investigator and two unbiased reviewers according to the following visual numeration: 0 = 0–10%; 1 > 10–25%; 2 > 25–50%; 3 > 50–75%; 4 > 75–100%, using a DM4000 microscope (Leica). OvCa tissues stained for HCAR1: brown indicating HCAR1 positive cellular components and blue/purple indicating haematoxylin counter stain. (**C**) HGSOC (stage I); (**D**) LGSOC (II); (**E**) CCC (III); (**F**) EAC (IV); (**G**) MAC (V); and (**H**) NAT. Images were captured using an Olympus BX51 light microscope at ×40 magnification.

A tissue microarray was also used to determine the protein expression of HCAR1 across a panel of different histological subtypes of epithelial OvCa and at different stages (I–IV; Figure 4A,B). Here, HCAR1 expression is widespread throughout the subtypes of OvCa, with varying intensity seen throughout different cell types. Representative images of the different histological subtypes are presented in Figure 4. Based on the staining in HGSOC and low-grade serous ovarian cancer (LGSOC) (Figure 4C,D), HCAR1 appears localised to the membrane of papillary cells with less intense (light brown) staining seen in the surrounding stroma. In cases of clear cell carcinoma (CCC), clear cytoplasmic regions are surrounded with connective trabeculae and show higher HCAR1 expression within glandular regions (Figure 4E). A similar pattern of high HCAR1 expression is detected in glandular epithelia (Figure 4F), in contrast to central stromal tissue in endometroid adenocarcinoma of the ovary (EAC). Intense (dark brown) staining is also seen amongst mucinous cystic epithelium of mucinous adenocarcinoma (MAC) compared with the surrounding layer of theca cells (Figure 4G). In normal adjacent tissues (NAT), the theca tissue, which encompasses primordial follicles, is surrounded by granulosa cells exhibiting high levels of HCAR1 (Figure 4H). Detailed review of intensity scores is presented in Supplementary Figure S2.

### 3.4. HCAR1 Expression Shows Little Influence on the Overall Survival (OS) or the Progression Free Survival (PFS) of Patients with OvCa

**Figure 5:**
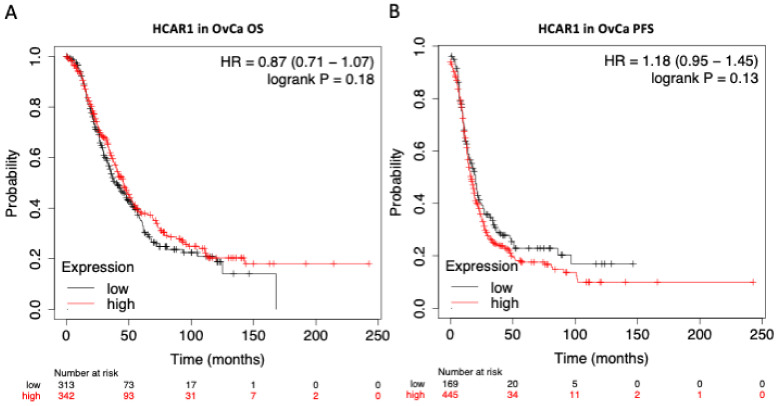
Ovarian Cancer (OvCa) Survival Curve analysis. Kaplan–Meier plots revealing the prognostic effects of HCAR1 expression in OvCa; (**A**) overall survival (OS); (**B**) progression-free survival (PFS). Generated through the Kaplan–Meier plotter (kmplot.com).

The rates of OS and PFS, in light of HCAR1 expression status (high vs. low), were assessed using the Kaplan–Meier plotter over a course of 250 months, using data acquired collectively through The Cancer Genome Atlas (TCGA), Gene Expression Omnibus (GEO), and the European Genome-Phenome Archive (EGA). Based on this in silico modelling, no overall difference can be noted for OS or PFS, regardless of HCAR1 expression level (Figure 5).

## 4. Discussion

In this study we demonstrate that resting lactate levels from circulating blood are significantly elevated in Stage III/IV OvCa patients compared to healthy controls. The increase noted in our patients, was independent of treatment, current cancer status (remission or recurrence) and BRCA status. Concomitant increased levels of mRNA for the lactate receptor HCAR1 in OvCa patients compared to controls were seen as well as widespread protein expression of this GPCR in OvCa patients.

Over the last decade, lactate has increasingly gained traction for its role as a signalling molecule and not just as a by-product of anaerobic cellular processes and glycolysis. The generation of lactate is thought to disrupt natural feedback mechanisms of normal cellular processes and promote metastasis and angiogenesis, thus contributing to poor prognosis in patients with cancer [21,37]. For example, lactate is thought to promote tumorigenesis by enhancing TGF-β signalling in regulatory T-cells. It also plays a role in the promotion of inflammation and angiogenesis [28].

Notably, although the normal range of circulating lactate at rest is 0.2 to 2.3 mmol/L, patients with cancer are shown to exhibit markedly higher levels [38]. We substantiate this in our present data, where patients with HGSOC exhibit levels with a median of 4.3 mmol/L, compared to 1.4 mmol/L in the control group. Of note, these patients did not present with typical symptoms of lactic acidosis, a condition characterised by high lactate levels and accompanying symptoms, such as muscle ache, nausea, breathing difficulties or stomach pain. These blood samples were instead taken as part of routine monitoring [39]. Similar results have been recorded in gliomas where interestingly, not only is there a difference between gliomas and normal glial tissue but patients with high-grade gliomas have significantly higher levels of resting lactate than those with low-grade gliomas, with lactate levels up to 14 mmol/L in the former [24]. Emerging data also support the potential of serum lactate as a biomarker of metastasis in brain tumours, while levels have also been studied in relation to bladder cancer using a urine liquid biopsy approach [24,25,26]. Moreover, retrospective data obtained from emergency department visits at a tertiary US hospital (lactate drawn from 1837 patients with various cancers and 3603 non-cancer patients) showed that, compared to non-cancer patients, cancer patients with elevated lactate levels exhibited a significantly increased risk of mortality [40]. Future studies are required to prospectively confirm such data and also further explore which bodily fluid encompasses the best liquid biopsy to measure lactate levels from.

This accumulation of resting lactate in patients with cancer, corresponds to an abnormally high metabolic rate and an increase in glycolytic processes within the cancer tissue [7]. Higher lactate may signify both an oxygen deficit within the patients’ tissues as well as a preference for the glycolytic pathway, both supporting the Warburg theory [41]. Contrary to the patients without cancer who have high lactate levels of around 9.0 ± 5.3 mmol/L in non-survivors, and around 3.4 ± 1.1 mmol/L in survivors (e.g., those with septic shock, myocardial infarction, respiratory distress, etc.), cancer patients are not as obviously sick and are able to undertake most activities of daily living [42,43].

As aforementioned, the elevated blood levels of lactate in patients with cancer presenting at the emergency department have been noted to negatively impact survival outcomes [40]. In our study, it was not possible to identify a prognostic role for lactate, as we acknowledge the limitation of a small cohort. Larger numbers of HGSOC OvCa patients will therefore provide a better understanding of lactate’s clinical utility in prognosis.

To further assess the signalling potential of circulating lactate in the OvCa tumour microenvironment, we explored expression of its cognate receptor, HCAR1, in normal and cancer tissues. Using in silico approaches, we confirmed the published widespread HCAR1 expression throughout an array of cancer types, including OvCa (Figure S1). It is intriguing to note that normal fallopian tube tissue and cervical tissue have higher HCAR1 expression than tissue from other areas of the female genital tract. Unsurprisingly, HCAR1 protein expression was observed across all histological subtypes of ovarian cancer, with localisation primarily around cellular membranes, in accordance with GPCR distribution. Widespread expression with dense staining was also identified in cells that are endocrine active, thus requiring increased energy, such as the granulosa and glandular cells, as well as the papillary type cells of HGSOC and LGSOC. Future studies are required to expand on these present findings, and also explore whether high levels of lactate cause any internalisation of HCAR1. Intense staining of granulosa cells surrounding the primordial follicle was also noted in NAT tissues. Although the over-expression of HCAR1 seen in NAT diverts from the expression trend seen at gene level in the control cohort for normal ovarian tissue, it should be recognised that the proximity of NAT has been shown to bear very similar characteristics to its adjacent malignant tissue, as preconditioning with transcriptional dysregulation may be underway [44].

Of note, lactate holds a complex role within the TME and is also associated with a suppression of innate immunity [45]. Given the rich metabolic state of immune cells, researchers have investigated the expression of HCAR1 in cells of the immune system, detecting HCAR1 expression in macrophages and dendritic cells. In macrophages elevated lactate increases activation of HCAR1 leading to the suppression of NF-kB pathways and subsequent reduction in cytokine production [46]. While in dendritic cells of mice with breast cancer, HCAR1 activation is also shown to reduce the production of IL-6 and IL-12. In addition, HCAR1 stimulation is seen to suppress MHC-II compromising tumour antigen presentation within T-cells preventing tumour recognition [47]. HCAR1 activation in breast cells, also appears to augment the expression of PD-L1 further aiding evasion of the immune system [48].

There are several limitations to this work. Ideally the control group should be a similar size and aged matched. Larger cohorts of OvCa patients, with earlier stage, disease would add confidence to the higher resting lactate levels seen in this group and give a better insight as to whether this is related to advanced disease only. Levels of lactate in patients with other histological subtypes of ovarian cancer such as clear cell, mucinous and low-grade should also be explored. Correlation of the resting blood lactate levels with HCAR1 protein expression in paired clinical samples (i.e., same OvCa patient) could yield information about the dynamics of lactate and its’ effects on patients with advanced OvCa. Moreover, it will be interesting to replicate the in silico experiments, particularly the ones on OS and PFS, using a substantial clinical cohort. Finally, further exploration of resting lactate levels and PD-1/PD-L1 expression on cancer cells and assessment of the T cell subsets in OvCa, together with a better understanding of the effect of lactate on macrophages in OvCa, may help us further understand why OvCa is resistant to immunotherapy.

## 5. Conclusions

The novel findings of the present study indicate that circulating lactate levels in patients with OvCa at rest are higher than normal in healthy female controls. This suggests that circulating lactate levels may hold screening potential for earlier detection of OvCa, prompting further diagnostic examinations. Circulating lactate levels are already regularly assessed in certain clinical settings, typically to assess the onset of sepsis. Although lactate can be measured routinely in patients, so far this approach has not been utilized to potentially assist cancer diagnosis, management or prognosis [37]. Given that lactate monitoring is a simple, cost-efficient, and readily available tool that can offer point-of-care results, the overall findings of the present study suggest that the potential of circulating lactate as a biomarker in OvCa merits further research attention. Thus, prospectively exploring the potential of lactate as an effective screening/prognostic biomarker to aid clinical practice in the fight against a common gynaecological cancer that is all too often characterized by late diagnosis and poor overall survival.

## Data Availability

Upon request from principal investigator.

## Supplementary Materials

The following supporting information can be downloaded at: https://www.mdpi.com/article/10.3390/jcm12010217/s1, Figure S1: Gene expression of HCAR1 (hydroxycarboxylic acid receptor 1, HCAR1; formerly known as GPR81) from The Cancer Genome Atlas (TCGA) in cancer and normal tissues. Figure S2: Bland–Altman analysis of immunohistochemistry quantification scores. Table S1: Details of tissue microarray.
