# Supplementary material for: Elevated Circulating Lactate Levels and Widespread Expression of Its Cognate Receptor, Hydroxycarboxylic Acid Receptor 1 (HCAR1), in Ovarian Cancer
Source: J Clin Med. 2022 Dec 27;12(1):217. doi: 10.3390/jcm12010217 (PMC9821497; doi:10.3390/jcm12010217)
Supplement: Supplementary file 1 [file jcm-12-00217-s001.zip › jcm-2043773-supplementary.pdf]

Supplementary Materials

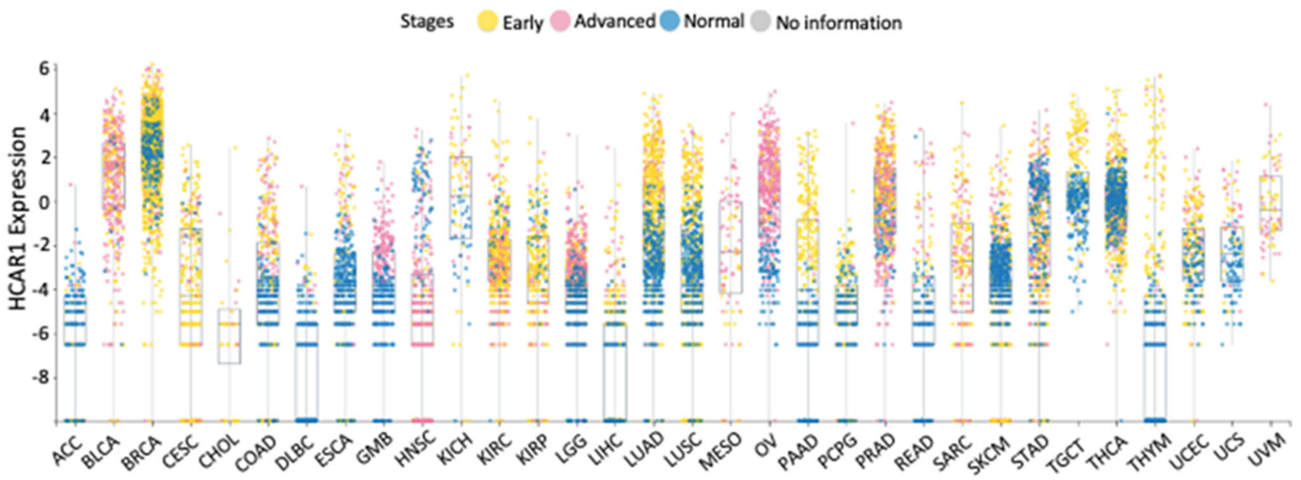

Figure S1. Gene expression of HCAR1 (hydroxycarboxylic acid receptor 1, HCAR1; formerly known as GPR81) from The Cancer Genome Atlas (TCGA) in cancer and normal tissues. Pancancer expression of HCAR1 taken from the canSAR database, showing expression from normal to early to advanced stages; Abbreviations—ACC: adrenocortical carcinoma; BLCA: bladder urothelial carcinoma; BRCA: breast invasive carcinoma; CESC: cervical squamous cell carcinoma and endocervical adenocarcinoma; CHOL: cholangio carcinoma; COAD: colon adenocarcinoma; DLBC: lymphoid neoplasm diffuse large B-cell lymphoma; ESCA: oesophageal carcinoma; GBM: glioblastoma multiforme; HNSC: head and neck squamous cell carcinoma; KICH: kidney chromophobe; KIRC: kidney renal clear cell carcinoma; KIRP: kidney renal papillary cell carcinoma; LAML: acute myeloid leukaemia; LGG: brain lower-grade glioma; LIHC: liver hepatocellular carcinoma; LUAD: lung adenocarcinoma; LUSC: lung squamous cell carcinoma; MESO: mesothelioma; OV: ovarian serous cystadenocarcinoma; PAAD: pancreatic adenocarcinoma; PCPG: pheochromocytoma and paraganglioma; PRAD: prostate adenocarcinoma; READ: rectal adenocarcinoma; SARC: sarcoma; SKCM: skin cutaneous melanoma; STAD: stomach adenocarcinoma; TGCT: testicular germ cell tumours; THCA: thyroid carcinoma; THYM: thymoma; UCEC: uterine corpus endometrial carcinoma; UCS: uterine carcinosarcoma; UVM: uveal melanoma. Expression measured as transcripts per million (TPM).

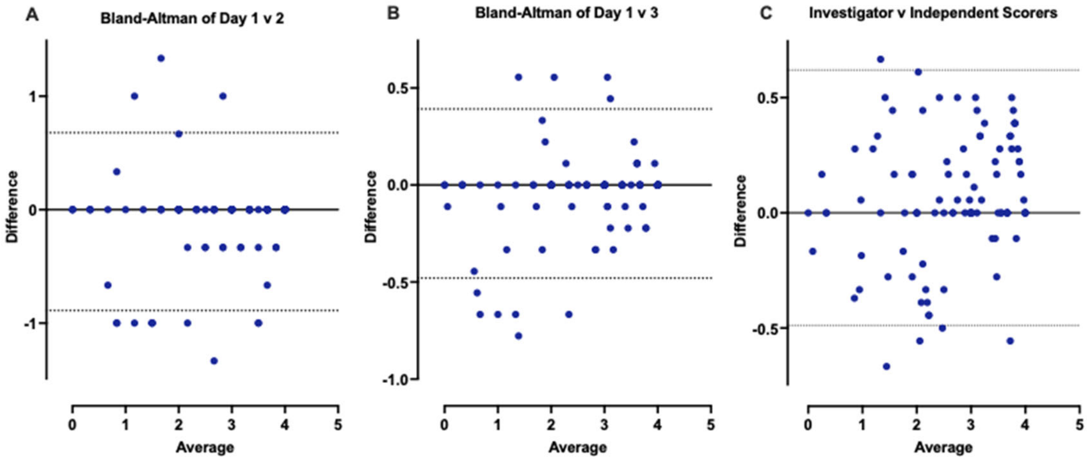

Figure S2. Bland–Altman analysis of immunohistochemistry quantification scores. (**A**) Primary investigator results for day 1 compared with day 2 of numeration, 95% confidence interval represented by the dashed line, each point represents an individual ovarian biopsy; (**B**) primary investigator results for day 1 compared to day 3; (**C**) comparison of quantification scores between primary investigator and independent reviewers for an average of 3 days, an insignificant amount of scores for biopsy cores are seen outside of the 95% confidence limit when data is pooled with independent reviewers.

Table S1. Patient details of tissue microarray.

Description	Ovary Cancer Tissue Array						
Cases	100						
Position	Age (years)	Sex	Organ/Anatomic Site	Pathology diagnosis	TNM	Stage	Type
A1	43	F	Ovary	Clear cell carcinoma	T1N0M0	I	Malignant
A2	61	F	Ovary	Clear cell carcinoma	T1aN0M0	IA	Malignant
A3	50	F	Ovary	Clear cell carcinoma	T1N0M0	I	Malignant
A4	40	F	Ovary	Clear cell carcinoma	T1N0M0	I	Malignant
A5	48	F	Ovary	Clear cell carcinoma	T2N0M0	II	Malignant
A6	50	F	Ovary	Low-grade serous carcinoma	T2aN0M0	IIA	Malignant
A7	60	F	Ovary	Low-grade serous carcinoma	T1cN0M0	IC	Malignant
A8	69	F	Ovary	Endometrioid adenocarcinoma	T1aN0M0	IA	Malignant
A9	41	F	Ovary	Low-grade serous carcinoma	T1N0M0	I	Malignant
A10	37	F	Ovary	Low-grade serous carcinoma	T1aN0M0	IA	Malignant
B1	25	F	Ovary	Low-grade serous carcinoma	T1N0M0	I	Malignant
B2	34	F	Ovary	Low-grade serous carcinoma	T1aN0M0	IA	Malignant
B3	59	F	Ovary	Low-grade serous carcinoma	T1aN0M0	IA	Malignant
B4	34	F	Ovary	Low-grade serous carcinoma	T1bN0M0	IB	Malignant
B5	56	F	Ovary	High-grade serous carcinoma	T2N0M0	II	Malignant
B6	22	F	Ovary	High-grade serous carcinoma	T2bN0M0	IIB	Malignant
B7	33	F	Ovary	High-grade serous carcinoma	T1N0M0	I	Malignant
B8	56	F	Ovary	High-grade serous carcinoma	T1N0M0	I	Malignant
B9	48	F	Ovary	High-grade serous carcinoma	T1N0M0	I	Malignant
B10	43	F	Ovary	High-grade serous carcinoma	T1N0M0	I	Malignant
C1	48	F	Ovary	High-grade serous carcinoma	T2bN0M0	IIB	Malignant
C2	51	F	Ovary	High-grade serous carcinoma	T3cN1M0	IIIC	Malignant
C3	42	F	Ovary	High-grade serous carcinoma	T1N0M0	I	Malignant
C4	47	F	Ovary	High-grade serous carcinoma	T3N0M0	III	Malignant
C5	64	F	Ovary	High-grade serous carcinoma	T1N0M0	I	Malignant
C6	52	F	Ovary	High-grade serous carcinoma	T1aN0M0	IA	Malignant
C7	53	F	Ovary	High-grade serous carcinoma	T2N0M1	IV	Malignant
C8	60	F	Ovary	High-grade serous carcinoma	T1bN0M0	IB	Malignant
C9	54	F	Ovary	High-grade serous carcinoma	T3cN1M0	IIIC	Malignant
C10	53	F	Ovary	High-grade serous carcinoma	T1N0M0	I	Malignant
D1	47	F	Ovary	High-grade serous carcinoma	T1aN0M0	IA	Malignant
D2	48	F	Ovary	High-grade serous carcinoma	T1N0M0	I	Malignant
D3	48	F	Ovary	High-grade serous carcinoma	T3cN0M0	IIIC	Malignant
D4	53	F	Ovary	High-grade serous carcinoma	T1cN0M0	IC	Malignant
D5	26	F	Ovary	High-grade serous carcinoma	T3cN1M0	IIIC	Malignant
D6	35	F	Ovary	High-grade serous carcinoma	T1aN0M0	IA	Malignant
D7	58	F	Ovary	High-grade serous carcinoma	T1N0M0	I	Malignant
D8	60	F	Ovary	High-grade serous carcinoma	T1aN0M0	IA	Malignant
D9	55	F	Ovary	High-grade serous carcinoma	T1N0M0	I	Malignant
D10	67	F	Ovary	High-grade serous carcinoma	T1aN0M0	IA	Malignant
E1	57	F	Ovary	High-grade serous carcinoma	T3cN1M0	IIIC	Malignant
E2	41	F	Ovary	High-grade serous carcinoma	T1N0M0	I	Malignant
E3	63	F	Ovary	High-grade serous carcinoma	T1N0M0	I	Malignant
E4	52	F	Ovary	High-grade serous carcinoma	T2N0M0	II	Malignant
E5	66	F	Ovary	High-grade serous carcinoma	T3N1M0	IIIC	Malignant
E6	52	F	Ovary	High-grade serous carcinoma	T1cN0M0	IC	Malignant
E7	64	F	Ovary	High-grade serous carcinoma	T3N1M1	IV	Malignant

E8	62	F	Ovary	High-grade serous carcinoma	T2N0M0	II	Malignant
E9	42	F	Ovary	High-grade serous carcinoma	T2N0M0	II	Malignant
E10	49	F	Ovary	High-grade serous carcinoma	T2N0M0	II	Malignant
F1	59	F	Ovary	High-grade serous carcinoma	T1cN0M0	IC	Malignant
F2	42	F	Ovary	High-grade serous carcinoma	T3cN1M0	IIIC	Malignant
F3	49	F	Ovary	High-grade serous carcinoma	T1aN0M0	IA	Malignant
F4	69	F	Ovary	High-grade serous carcinoma	T2N0M0	II	Malignant
F5	42	F	Ovary	High-grade serous carcinoma	T3N1M0	IIIC	Malignant
F6	53	F	Ovary	High-grade serous carcinoma	T1aN0M0	IA	Malignant
F7	47	F	Ovary	High-grade serous carcinoma	T1cN0M0	IC	Malignant
F8	49	F	Ovary	High-grade serous carcinoma	T3N1M0	IIIC	Malignant
F9	52	F	Ovary	High-grade serous carcinoma (sparse)	T3cN1M0	IIIC	Malignant
F10	55	F	Ovary	High-grade serous carcinoma	T3cN1M1	IV	Malignant
G1	52	F	Ovary	High-grade serous carcinoma	T2N0M0	II	Malignant
G2	51	F	Ovary	High-grade serous carcinoma	T2N0M0	II	Malignant
G3	41	F	Ovary	High-grade serous carcinoma	T1aN0M0	IA	Malignant
G4	56	F	Ovary	High-grade serous carcinoma with necrosis	T3N0M0	III	Malignant
G5	55	F	Ovary	High-grade serous carcinoma	T1N0M0	I	Malignant
G6	48	F	Ovary	High-grade serous carcinoma	T3aN0M0	IIIA	Malignant
G7	60	F	Ovary	High-grade serous carcinoma	T2bN0M0	IIB	Malignant
G8	51	F	Ovary	High-grade serous carcinoma with necrosis	T1aN0M0	IA	Malignant
G9	46	F	Ovary	Mucinous papillary adenocarcinoma	T1aN0M0	IA	Malignant
G10	49	F	Ovary	Endometrioid adenocarcinoma	T1N0M0	I	Malignant
H1	34	F	Ovary	Mucinous adenocarcinoma	T1bN0M0	IB	Malignant
H2	37	F	Ovary	Mucinous adenocarcinoma	T1aN0M0	IA	Malignant
H3	39	F	Ovary	Mucinous adenocarcinoma with necrosis	T1aN0M0	IA	Malignant
H4	54	F	Ovary	Mucinous adenocarcinoma	T2aN0M0	IIA	Malignant
H5	41	F	Ovary	Mucinous adenocarcinoma	T1bN0M0	IB	Malignant
H6	50	F	Ovary	Mucinous adenocarcinoma with necrosis	T3cN1M0	IIIC	Malignant
H7	52	F	Ovary	Mucinous adenocarcinoma	T1bN0M0	IB	Malignant
H8	38	F	Ovary	Mucinous adenocarcinoma	T1N0M0	I	Malignant
H9	29	F	Ovary	Mucinous adenocarcinoma	T3N0M0	III	Malignant
H10	58	F	Ovary	Endometrioid adenocarcinoma	T1aN0M0	IA	Malignant
I1	47	F	Lymph node	Metastasis serous carcinoma from ovary	-	-	Metastasis
I2	48	F	Lymph node	Metastasis serous carcinoma from ovary	-	-	Metastasis
I3	46	F	Lymph node	Metastasis serous carcinoma from ovary	-	-	Metastasis
I4	54	F	Lymph node	Metastasis serous carcinoma from ovary	-	-	Metastasis
I5	83	F	Lymph node	Metastasis serous carcinoma from ovary	-	-	Metastasis
I6	48	F	Lymph node	Metastasis clear cell carcinoma from ovary	-	-	Metastasis
I7	56	F	Fibrofatty tissue	Metastasis serous carcinoma from ovary	-	-	Metastasis

I8	57	F	Greater omentum	Metastasis serous carcinoma from ovary	-	-	Metastasis
I9	50	F	Lymph node	Metastasis serous carcinoma from ovary	-	-	Metastasis
I10	53	F	Fibrofatty tissue	Metastasis serous carcinoma from ovary	-	-	Metastasis
J1	69	F	Ovary	Adjacent normal ovary tissue	-	-	NAT
J2	48	F	Ovary	Adjacent normal ovary tissue	-	-	NAT
J3	53	F	Ovary	Adjacent normal ovary tissue	-	-	NAT
J4	42	F	Ovary	Adjacent normal ovary tissue	-	-	NAT
J5	42	F	Ovary	Adjacent normal ovary tissue	-	-	NAT
J6	40	F	Ovary	Adjacent normal ovary tissue	-	-	NAT
J7	59	F	Ovary	Adjacent normal ovary tissue	-	-	NAT
J8	42	F	Ovary	Adjacent normal ovary tissue	-	-	NAT
J9	35	F	Ovary	Adjacent normal ovary tissue	-	-	NAT
J10	45	F	Ovary	Adjacent normal ovary tissue	-	-	NAT
